# Submesoscale coupling of krill and whales revealed by aggregative Lagrangian coherent structures

**DOI:** 10.1098/rspb.2023.2461

**Published:** 2024-02-21

**Authors:** James A. Fahlbusch, David E. Cade, Elliott L. Hazen, Meredith L. Elliott, Benjamin T. Saenz, Jeremy A. Goldbogen, Jaime Jahncke

**Affiliations:** ^1^ Hopkins Marine Station, Oceans Department, Stanford University, Pacific Grove, CA, USA; ^2^ Cascadia Research Collective, Olympia, WA, USA; ^3^ Ecosystem Science Division, NOAA Southwest Fisheries Science Center, Monterey, CA, USA; ^4^ California Current Group, Point Blue Conservation Science, Petaluma, CA, USA; ^5^ Department of Biology, University of South Florida, Tampa, FL, USA

**Keywords:** krill, cetaceans, california current system, physical–biological coupling, Lagrangian coherent structures, finite-time Lyapunov exponent (ftle)

## Abstract

In the marine environment, dynamic physical processes shape biological productivity and predator–prey interactions across multiple scales. Identifying pathways of physical–biological coupling is fundamental to understand the functioning of marine ecosystems yet it is challenging because the interactions are difficult to measure. We examined submesoscale (less than 100 km) surface current features using remote sensing techniques alongside ship-based surveys of krill and baleen whale distributions in the California Current System. We found that aggregative surface current features, represented by Lagrangian coherent structures (LCS) integrated over temporal scales between 2 and 10 days, were associated with increased (a) krill density (up to 2.6 times more dense), (b) baleen whale presence (up to 8.3 times more likely) and (c) subsurface seawater density (at depths up to 10 m). The link between physical oceanography, krill density and krill–predator distributions suggests that LCS are important features that drive the flux of energy and nutrients across trophic levels. Our results may help inform dynamic management strategies aimed at reducing large whales ship strikes and help assess the potential impacts of environmental change on this critical ecosystem.

## 1. Background

Unraveling the mechanisms that govern the abundance and distribution of predators and prey is fundamental to understanding the functioning of marine ecosystems. The dynamic nature of the ocean, and the physical–biological coupling that results, is thought to influence the occurrence of marine species [1]. Such biophysical interactions give rise to heterogeneously distributed resources, or patchiness across multiple and often hierarchical scales [2–4]. Patchiness influences the ability for primary producers (e.g. phytoplankton) to flourish, which in turn affects prey availability at higher trophic levels, from secondary consumers to top predators [5]. To build a mechanistic understanding of patchiness and how animals interact with their environment, interdisciplinary approaches should integrate information on physical processes and community structure at relevant spatiotemporal scales [1,6].

Patchily distributed prey impacts predator foraging efficiency by increasing the time and energy required to locate and consume prey [7]. Higher patch densities result in a greater number of available prey items within a given area, increasing the opportunities for predators to feed and meet their energetic demands. For example, locally dense areas of fish prey within a prey patch can be more strongly correlated with predator (i.e. seabird and pinniped) foraging behaviour than total available biomass of the patch [8]. This finding probably extends to krill predators in seasonally productive upwelling ecosystems like the California Current System (CCS), especially bulk filter feeding whales that rely on dense patches to increase foraging efficiency [9]. While blue whales (*Balaenoptera musculus*) primarily rely on krill as their main food source, humpback whales (*Megaptera novaeangliae*) in the CCS can switch between feeding on krill and schools of fish [10–13]. For blue whales, finding high density prey patches is particularly important to optimize foraging efficiency and fitness, given the constraints of their large body size [14]. Krill density may also influence the feeding preferences of generalist predators like humpback whales, potentially triggering a switch to fish as a food source at lower krill densities [12,13].

The CCS is characterized by the wind-driven upwelling of cold, dense, nutrient-rich waters from below the mixed-layer to the euphotic zone [15] providing the foundation of the CCS food web by promoting the growth of phytoplankton and subsequently krill (order *Euphausiacea*) [16,17]. Coastal upwelling in the CCS varies seasonally, with peak upwelling occurring during the spring and summer months as a result of predominantly alongshore, equatorward winds [18,19]. Within the upwelling season, pulsed wind events lead to smaller-scale upwelling–relaxation cycles that contribute to the overall variability of circulation in the CCS [20,21]. Circulation patterns interact with the irregular coastline as well as underwater topographic features to create a complex, dynamic environment at fine to broad scales [22].

Krill plays a pivotal role in connecting physical processes, primary production and higher trophic-level species as a key component of the CCS food web [23,24]. While krill distributions are not completely dictated by currents, having swimming speeds of approximately 5–10 cm s^−1^, they are influenced by physical forcing through the movements of water masses [25,26] and aggregative processes [21]. Additionally, krill aggregates into ephemeral swarms as a means of predator avoidance that are exploited by gulping predators like baleen whales [9,17,21]. The use of hydroacoustics surveys allows for fine-scale measurement of the horizontal and vertical distribution of krill over broad spatial areas [27,28]. Multi-year studies of krill in the CCS have used hydroacoustics to model persistent krill hotspots, which tend to occur downstream of upwelling centers [29,30] and along continental shelf-breaks or submarine canyon systems [29,31]. However, given environmental variability (seasonal and annual), and the time lag between primary production and secondary consumer (e.g. krill) growth, there is variability in the phenology and patchiness of krill in the CCS [24,32]. A regional study in the Central CCS found that most krill aggregations are short-lived (e.g. 2–10 days, 18 to 800 km^2^) and that large, persistent krill aggregations (greater than 20 days, 800–1000 km^2^) are rare [32]. These daily and sub-daily cycles of patch density have been linked to environmental fluctuations, including aggregative surface current features, and can be associated in space and time with aggregations of krill predators [21,33]. The short lifespan and fine-scale heterogeneity of these important prey patches underscore the importance of submesoscale (less than 100 km) biophysical relationships as mechanistic drivers of marine hotspot formation [34].

There is growing evidence that submesoscale physical processes play a critical role in the structuring of pelagic ecosystems, especially in the context of predator–prey interactions [35–37]. Recent research has delved into the relationships between pelagic predator habitat selection and surface current features (e.g. fronts and eddies), for pinnipeds [38], sharks [39,40], turtles [41], seabirds [37,42] and cetaceans [43–45]. These surface current features reflect processes that affect productivity through the transport of nutrient-rich water [46] and aggregation of organisms operating at low (10^−2^) and intermediate (10^2^–10^3^) Reynolds numbers through physical forcing [37]. The movement of water masses such as upwelling jets, eddies, and fronts, can affect the transport of phytoplankton and can contribute to the formation of krill hotspots [47–49]. However, a more complete understanding of these mechanisms is limited by our ability to contemporaneously measure ocean features and the distribution of krill patches at comparable scales [17,50].

Here we integrate physical and biological measurements from *in-situ* ship-based surveys with remotely sensed habitat features to investigate the drivers of krill density and distribution in the CCS. We use hourly high-frequency (HF) radar surface current measurements to calculate a time-dependent Lagrangian modelled proxy that reflects coherent aggregative ocean transport features at submesoscales [51,52]. Blue whales in the CCS have been shown to increase feeding rates at aggregative surface current features [45], suggesting that these features may correspond to increased krill density, but the mechanism by which these features would influence krill distributions has not been explained. We hypothesize that the surface current features identified as important to krill predators will have (1) both a surface and subsurface oceanographic expression, and (2) either increased krill abundance or density. Identifying these features in the ocean both climatologically and contemporaneously will help inform how best to monitor, predict and conserve predator hotspots in the future.

## 2. Material and methods

All data processing and statistical analyses were conducted in R v. 4.0.0 [53].

### (a) *In situ* data

**Figure 1.**
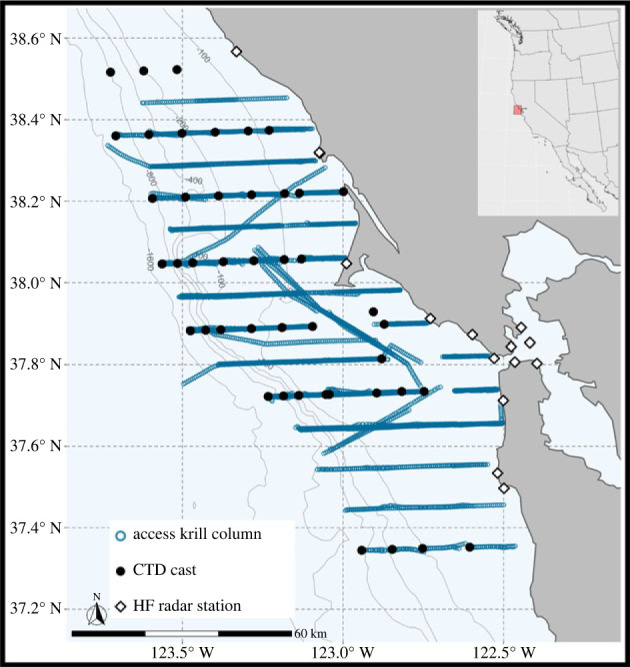
Map of ACCESS cruise transect lines 2012–2018 with markers depicting CTD stations (black circles) and HF Radar Stations (black and white diamonds). Isobaths are shown in light grey. Inset shows the bounding box (red square) of the HF Radar data used in this study. (Note: HF Radar coverage extends beyond the spatial extent of this map).

This study focused on the central California region of the CCS (37.2° N to 38.6° N, 122.2° W to 124° W), which is a coastal region comprising continental shelf and shelf-break habitat. Survey data for this study were collected by the Applied California Current Ecosystem Studies (ACCESS) program (www.accessoceans.org). Transect surveys were conducted across three seasons each year (spring, summer and autumn), and our analysis has been limited to the years 2012–2018 to overlap with remotely sensed data coverage in the region. Survey transect lines generally run along latitudinal parallels spanning the Greater Farallones and Cordell Bank National Marine Sanctuaries in Central California (figure 1 and table 1), collecting cetacean sighting locations, surface and water-column oceanographic parameters, zooplankton sampling, as well as hydroacoustics to estimate krill abundance (see [54,55] for additional details of the survey methods, raw data collection and processing procedures). To minimize the influence of diel patterns of vertical changes in krill distribution, we limited our analysis to include only survey data from daytime hours.

**Table 1.** Summary of ACCESS cruise survey effort between 2012 and 2018. Survey transects collected hydroacoustics data, cetacean sightings and periodic CTD profiles at fixed locations along the transect.

cruise ID	start date	end date	cruise duration (days)	no. transect lines	no. CTD profiles	cruise transect distance (km)
ACC1206	19/6/2012	24/6/2012	5	11	18	220.6
ACC1207	24/7/2012	29/7/2012	5	11	18	269.2
ACC1209	13/9/2012	19/9/2012	6	16	19	384.2
ACC1305	25/5/2013	30/5/2013	5	11	13	239.2
ACC1307	24/7/2013	30/7/2013	6	13	0	376.1
ACC1309	26/9/2013	1/10/2013	5	9	15	261.4
ACC1406	21/6/2014	26/6/2014	5	3	9	129.2
ACC1407	17/7/2014	24/7/2014	7	16	20	588.7
ACC1409	20/9/2014	27/9/2014	7	12	25	357.1
ACC1506	21/6/2015	26/6/2015	5	4	8	109.5
ACC1507	19/7/2015	25/7/2015	6	13	24	273.6
ACC1509	23/9/2015	25/9/2015	2	3	15	83.1
ACC1605	15/5/2016	22/5/2016	7	19	30	487.5
ACC1607	18/7/2016	22/7/2016	4	10	13	224.9
ACC1609	18/9/2016	21/9/2016	3	8	13	193.5
ACC1705	26/5/2017	29/5/2017	3	7	15	181.4
ACC1707	22/7/2017	28/7/2017	6	15	0	332.1
ACC1709	23/9/2017	29/9/2017	6	14	19	358.0
ACC1805	25/5/2018	29/5/2018	4	9	14	177.0
ACC1807	4/7/2018	10/7/2018	6	28	33	771.6
ACC1809	21/9/2018	28/9/2018	7	16	22	420.8

#### (i) Oceanography

*In situ* oceanographic observations were recorded using a Sea-Bird Electronics SBE 19Plus Conductivity-Temperature-Depth (CTD) profiler at permanent sampling stations along the survey transect lines (*n* = 343 CTD casts). The CTD sampled temperature and salinity vertically, from which an estimate of potential seawater density (sigma-theta, *σ_θ_*) was calculated at 1 m increments between the surface and maximum cast depth using the swSigmaTheta function (oce package, v1.7.2 [56]) in R. Potential density is the estimated density that seawater would have if raised adiabatically to the surface and allows for the comparison of the density of water masses at different depths without the influence of pressure on seawater density [57]. To allow for comparability across samples, we excluded CTD profiles with a maximum cast depth of less than 50 m.

#### (ii) Krill presence and density

Zooplankton data were previously published in Manugian *et al.* [54] and Rockwood *et al.* [55]). Briefly, data were sampled acoustically every 2 s along transect survey lines using Simrad EK60 split beam echosounders at 38 and 120 kHz with 1024 µs pulse lengths. Acoustic data were integrated to produce average backscatter in 200 m horizontal by 5 m vertical grid cells. Depths less than 5 m were not analysed to avoid surface interference. Ship echosounder systems were calibrated annually prior to spring surveys. A dB difference (120 kHz – 38 kHz) of 8–23 dB was used to identify krill swarms in the integrated backscatter cells, and backscatter data were then processed to estimate the average density of krill (grams of krill/m^3^) using cruise-specific target strengths derived from the stochastic distorted-wave Bourne approximation for Antarctic krill [58] using lengths of krill (8–30 mm) sampled during each cruise (electronic supplementary material, table S1). It should be noted that biomass in 200 m horizontal by 5 m vertical grid cells is meant to be representative of relative prey availability in an area, not necessarily what would be consumed by a predator targeting specific parts of a patch [8,59]. Similarly, updated target strength models for NE Pacific krill (e.g. [59] and [60]) would not affect our model results.

**Figure 2.**
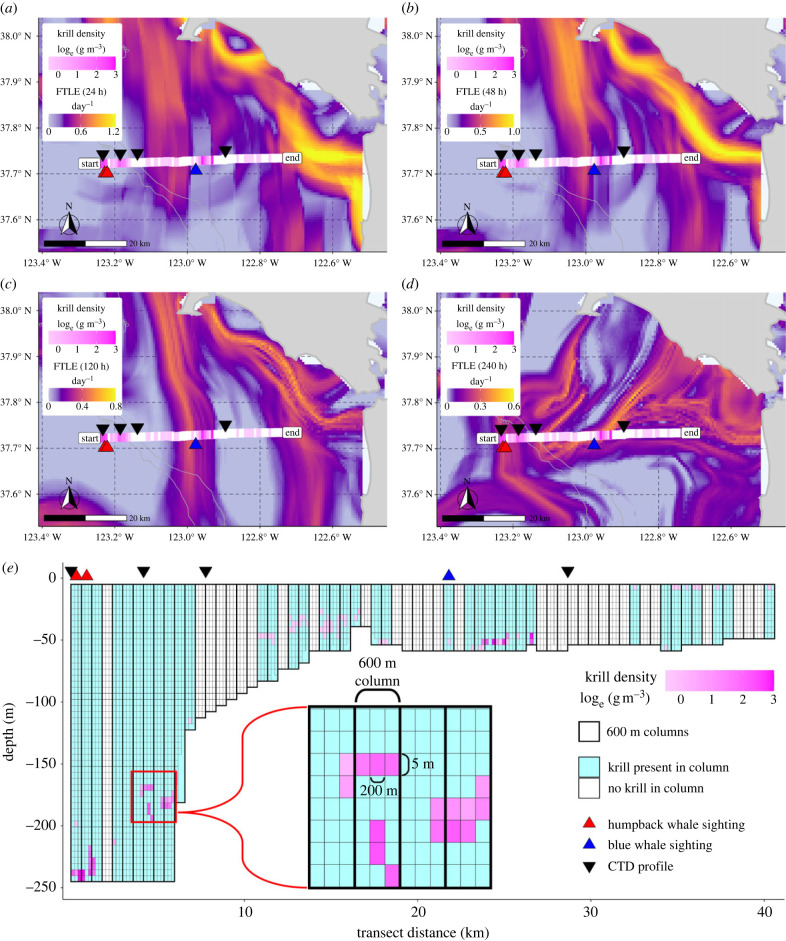
Spatial distribution of FTLE, krill density, cetacean sightings and CTD profile locations for a representative transect line in July of 2012. (*a–d*) Mean FTLE of the transect line time-period for the 24, 48, 120 and 240 h integration windows, respectively. Krill density (magenta colour-ramp) is the logged mean krill density of grid cells within each 600 m column (white denotes no krill present). (*e*) Vertical distribution of krill within 600 m horizontal, full depth columns (each composed of 200 m horizontal by 5 m vertical grid cells; see inset) along the same transect line shown in the panels above. Black vertical lines denote the 600 m columns which are shaded by krill presence (light blue shading) and absence (white shading) within the column. Krill density (magenta colour-ramp) is the logged krill density for 200 m horizontal by 5 m vertical grid cells with krill present. Triangle markers in all panels denote the locations of humpback (red) and blue (blue) whale sightings, and the locations of CTD profiles (black) along the transect.

For each survey transect line, 200 m horizontal by 5 m vertical grid cells were integrated into 600 m horizontal, full depth columns (hereafter 600 m columns) to match the spatial resolution of the remotely sensed metrics used in our analysis (figure 2). End-of-line bins (less than 600 m in length) were discarded to avoid bias. Krill presence was assigned for each column if any of the 200 m horizontal by 5 m vertical grid cells within a 600 m column contained krill. For columns that contained krill, we calculated several metrics to quantify krill density within the column for our analysis. The distribution of krill in our dataset is gamma distributed and as a result, we calculated the geometric mean krill density of cells containing krill (geometric mean of non-zero krill density) as the primary metric of krill density in our analysis. For comparison, we also calculated the arithmetic mean krill density of cells containing krill (mean of non-zero krill density) and the maximum krill density within a column (max of non-zero krill density).

#### (iii) Cetacean sightings

Visual cetacean sightings were recorded in conjunction with the krill transect surveys using standardized line-transect methods [61] with one observer on each side and a central line observer during daylight hours while the vessel was underway. To reduce potential bias in location accuracy of sightings, we excluded sightings further than 0.5 nautical miles from the ship (approx. 925 m) as well as sightings where the species could not be verified. Additionally, due to the low sample size of fin (*n* = 8 sightings), grey (*n* = 5 sightings) and minke (*n* = 2 sightings) whales, they are excluded from our analysis. The resulting dataset included 140 blue whale sightings (79% of total blue whale sightings) and 750 humpback whale sightings (84% of total humpback whale sightings) over the 7-year study period. Sighting presence or absence for each species were assigned to 600 m bins along krill transect lines to align with the spatial resolution of the remotely sensed metrics used in our analysis.

### (b) Remotely sensed data

ACCESS surveys overlapped with the sampling footprint of the US Integrated Ocean Observing System High Frequency (HF) Radar Network (IOOS HFRNet, [62]), which provides continuous, high-resolution measurements of ocean circulation and structure at fine and intermediate scales [63,64] In this study, HF Radar surface current vectors are used to calculate the backward-in-time, finite-time Lyapunov exponent (hereafter FTLE), which is a scalar measure of the rate of attraction of simulated particle tracers advected using empirically measured surface current flows. Ridges of elevated FTLE values identify Lagrangian coherent structures (LCS), which represent barriers to transport, such as fronts and eddies [65] and have been linked to krill predator movement and foraging behaviour in the same region [33,43,45].

FTLE is a measure of the sensitivity of fluid particle trajectories to initial conditions over a given time period (i.e. integration window), providing information about the stretching and folding of fluid elements in a flow field [66]. The integration duration refers to the length of time over which the fluid particle trajectories are computed. Short integration durations capture the local behaviour of the flow field, highlighting small-scale features and rapid changes in the flow, which is useful for examining details of processes occurring on shorter time scales. Longer integration durations capture the cumulative effects of advection and other processes over a longer time period, allowing for the detection of larger-scale flow patterns and coherent structures that may not be evident in short-term analyses [65].

For each cruise, surface current data were downloaded from IOOS HFRNet (http://cordc.ucsd.edu/projects/hfrnet/) at hourly resolution (cells 6 km on a side) for the period ±14 days of each cruise with a common bounding box of ±1 degree around the survey locations for all cruises (bounding box shown in the inset of figure 1). Data gaps in the raw HF Radar surface current measurements were restored (*sensu* [67]) and FTLE was calculated using trajectory reconstruction and analysis for coherent structure evaluation (TRACE; http://transport.me.berkeley.edu/trace) [68–70] following the same processing steps used in [45] for identifying Lagrangian habitat features. To evaluate the scale of ecological processes and dynamical features influencing the relationships in our study (e.g. krill distribution and density), we selected 4 FTLE integration windows (24, 48, 120 and 240 h). At every hourly time-step, the simulated trajectories of an evenly spaced grid of tracers were integrated over the time period corresponding to each integration window and FTLE was calculated from the time-dependent movement of tracer trajectories. It should be noted that there is overlap in the calculation of FTLE for all integration windows as all calculations have the same set of starting timestamps. For example, the surface current flows influencing the 24 h integration are the same as the initial 24 h for the corresponding 48, 120 and 240 h integrations with the same start time. All FTLE calculations use the same grid of tracers and have the same temporal and spatial resolution for all integration windows (figure 2*a–d*).

FTLE is calculated at hourly intervals, with each FTLE value representing ±30 min from the sample timestamp. Most survey transect lines spanned multiple FTLE measurement increments (mean = 3.02 h). We calculated a spatial mean of the FTLE layers for each transect line, and extracted the FTLE value for the midpoint location of each 600 m column for both the krill and cetacean sightings. Because the CTD profiles were sampled periodically, we extracted the FTLE values for the specific time and location of the CTD profile. FTLE data extraction and layer processing used the raster package [71] in R.

### (c) Statistical analyses

Our analyses include generalized linear mixed models (GLMMs) to test whether remotely sensed aggregative surface current features (areas of elevated FTLE) correspond to changes in oceanographic parameters, krill presence and density, and cetacean presence. Each GLMM in our study includes year, season and transect line as nested random effects to account for spatial and temporal variation among survey efforts. Additionally, to determine the scale of process influencing each parameter, we fitted a separate model for each of the 4 integration windows (24, 48, 120 and 240 h) with FTLE as the predictor variable. Model assumptions were checked visually using diagnostic plots and model residuals were tested for autocorrelation.

#### (i) Oceanographic expression of FTLE

To test whether remotely sensed aggregative surface current features (i.e. areas of elevated FTLE) correspond to *in situ* oceanographic features, we fitted a linear regression (glmer function of the lme4 package, v1.1-27.1 [72]) with potential seawater density (*σ_θ_*) as the response variable. We performed this analysis at 6 different depths (0, 10, 20, 30, 40 and 50 m) to determine whether the relationship between seawater density and FTLE is depth-dependent. To further explore the depth of influence of aggregative surface current features, we also fit a linear regression (glmer function of the lme4 package, v1.1-27.1 [72]) with the depth of potential seawater density (*σ_θ_*) at 25, 25.5 and 26 kg m^–3^ as the response variable.

#### (ii) Krill presence and density

To examine the probability of krill presence across a range of FTLE values, we fitted a logistic regression using the binomial family and a logit-link (glmmPQL function of the MASS package, v7.3-53 [73]) with krill presence or absence within 600 m columns as the response variable. We incorporated an exponential autocorrelation structure (corExp, nlme package v3.1-147 [74]) using the midpoint latitude and longitude of each 600 m column as a covariate to account for spatial autocorrelation in our model.

To explore the relationship between krill aggregation and FTLE, we fitted a total of 12 linear regressions (glmmPQL) with each using one of three metrics of krill density (geometric mean of non-zero krill density, mean of non-zero krill density, maximum of non-zero krill density) as the response variable across 4 temporal scales. For these analyses, we are only testing the influence of FTLE on 600 m columns that contain krill. Krill density is gamma distributed (positively skewed) in our dataset and we fitted the models using the gamma family with a log-link in which the coefficients for the fixed effects are the log-transformed odds ratio of the response variable associated with a one-unit increase in the predictor variable. We incorporated an exponential autocorrelation structure (corExp, nlme package v3.1-147 [74]) using the midpoint latitude and longitude of each 600 m column as a covariate to account for spatial autocorrelation.

#### (iii) Cetacean presence

To assess whether cetaceans are more likely to be sighted in areas of elevated FTLE, we fitted a logistic regression (glmmPQL) with whale presence or absence as the response variable. Sighting probability was modeled using a logit-link function in the binomial family, and the coefficients for the fixed effects are the log-transformed odds ratio of the response variable associated with a one-unit increase in the predictor variable. Each species was modeled separately and model residuals were checked for autocorrelation.

## 3. Results

We examined the influence of aggregative surface current features on oceanographic parameters, krill patchiness and cetacean presence across a range of temporal scales using transect survey data spanning 7 years in the Central California region (*n* = 240 transect lines). Our analysis found significant associations between FTLE features and seawater density (extending to a depth of 10 m), krill density and cetacean presence. Additionally, our results show a scale-dependency in each of these associations, and we examine each of them in further detail below.

### (a) Oceanographic expression of FTLE

The relationship between FTLE and seawater density is both depth and scale dependent. We found a significant, positive relationship between seawater density and FTLE at both the surface and at 10 m for the 120 h integration window, and while not significant, the slopes for all other integration windows were positive for the surface and 10 m depths (figure 1*a*). The relationship changes as depth increases beyond 10 m. For depths of 20–50 m, the coefficients for the 24* and 48 h integration windows are negative (table 2; electronic supplementary material, figure S1). For the 120 and 240 h integration windows, the coefficients of models for depths of 20–50 m are all positive though not significant, and follow a pattern diminishing slope estimate with increasing depth. Coefficients for all modelled depths followed a similar pattern, generally increasing with increasing integration times with a peak at 120 h and a slight decrease for the 240 h integration window. Our analysis of the relationship between FTLE and the depth of specific values of potential sweater density supported the findings above (see electronic supplementary material, figure S2). For example, the depth of the 25.5 kg m^–^^3^ isopycnal had a significant negative relationship with FTLE for the 120 and 240 h integration windows, meaning that there was shoaling in areas of elevated FTLE.

**Table 2.** Model results at each of the 4 integration windows (24, 48, 120 and 240 h). Bold values indicate models where the predicted slope was significantly different from 0 (i.e. *p*-value < 0.05).

model response variable	24 h			48 h			120 h			240 h		
	slope	intercept	*p*-value	slope	intercept	*p*-value	slope	intercept	*p*-value	slope	intercept	*p*-value
potential seawater density (*σ_θ_*) at surface	0.05	25.20	0.525	0.13	25.19	0.190	**0.37**	**25**.**23**	**0**.**001**	0.29	25.26	0.051
potential seawater density (*σ_θ_*) at 10 metres	0.02	25.36	0.844	0.12	25.34	0.199	**0**.**34**	**25**.**37**	**0**.**002**	0.25	25.40	0.068
potential seawater density (*σ_θ_*) at 20 metres	**−0**.**17**	**25**.**60**	**0**.**015**	−0.03	25.57	0.668	0.16	25.58	0.066	0.16	25.62	0.142
potential seawater density (*σ_θ_*) at 30 metres	**−0**.**17**	**25**.**78**	**0**.**004**	−0.08	25.76	0.227	0.08	25.77	0.317	0.14	25.79	0.154
potential seawater density (*σ_θ_*) at 40 metres	**−0**.**14**	**25**.**88**	**0**.**009**	−0.04	25.86	0.495	0.09	25.87	0.190	0.14	25.89	0.126
potential seawater density (*σ_θ_*) at 50 metres	**−0**.**13**	**25**.**97**	**0**.**005**	−0.03	25.95	0.592	0.07	25.96	0.264	0.10	25.98	0.215
krill presence	**−0**.**38**	**0**.**08**	**0**.**014**	−0.27	0.02	0.137	0.26	−0.13	0.290	0.29	−0.17	0.370
mean krill density of non-zero cells	0.21	1.16	0.188	**0**.**44**	**1**.**11**	**0**.**017**	**0**.**93**	**1**.**10**	**0**.**000**	**0**.**97**	**1**.**12**	**0**.**002**
geometric mean krill density of non-zero cells	0.27	0.82	0.084	0.36	0.82	0.056	**0**.**82**	**0**.**82**	**0**.**001**	**0**.**97**	**0**.**83**	**0**.**003**
maximum krill density of non-zero cells	0.07	1.97	0.686	**0**.**41**	**1**.**89**	**0**.**040**	**0**.**88**	**1**.**83**	**0**.**001**	**0**.**83**	**1**.**81**	**0**.**016**
blue whale presence	**0**.**79**	**−5**.**92**	**0**.**007**	**1**.**34**	**−5**.**99**	**0**.**000**	**1**.**20**	**−5**.**83**	**0**.**010**	**2**.**12**	**−5**.**98**	**0**.**001**
humpback whale presence	**0**.**36**	**−3**.**63**	**0**.**036**	**0**.**45**	**−3**.**61**	**0**.**030**	−0.43	−3.48	0.138	0.63	−3.60	0.101

### (b) Krill presence and density

The results of the logistic regression for krill presence indicate that krill is not more likely to be found in areas of elevated FTLE for any of the 4 integration windows. Krill was present in 50.6% of the columns analysed. We did observe a significant negative relationship between FTLE and krill presence for the 24 h integration time (table 2). Similar to the results for seawater density, the coefficients for each model increased with increasing integration time.

**Figure 3.**
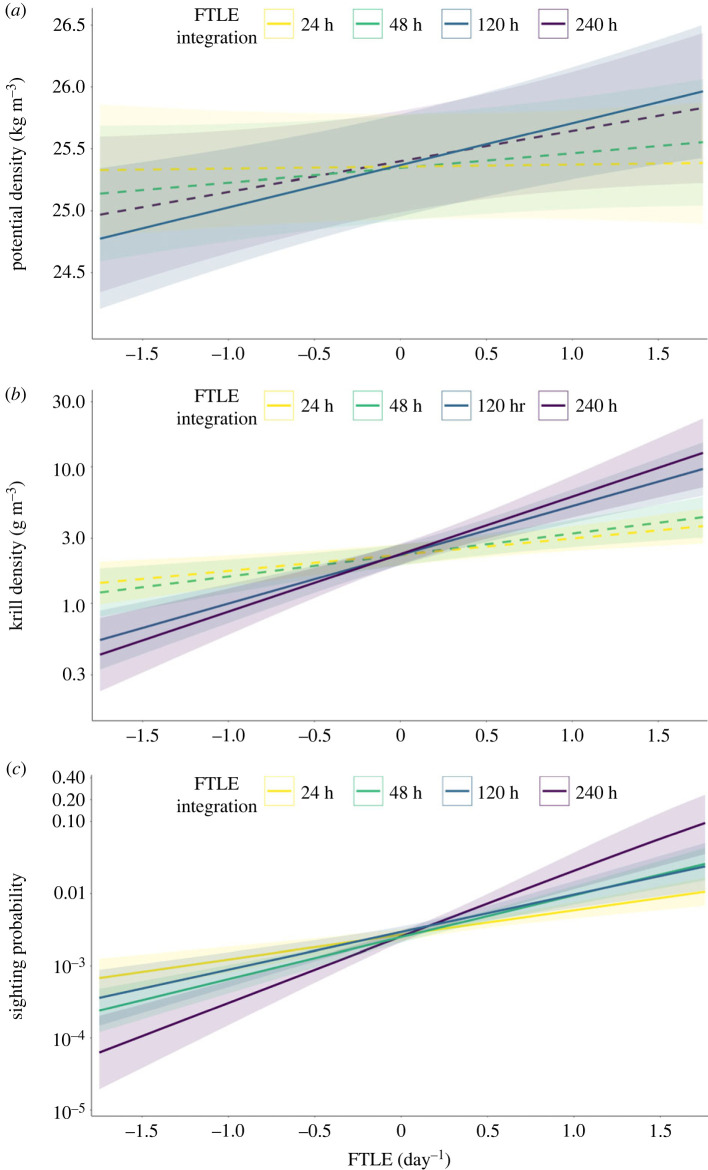
Predicted relationships between FTLE at each of four integration windows and values of (*a*) seawater potential density at 10 m, (*b*) krill density (geometric mean density of cells containing krill in a column) and (*c*) blue whale sighting probability. Colour scheme denotes FTLE integration durations of 24 h (yellow), 48 h (green), 120 h (blue) and 240 h (purple), which is used as a proxy for the scale of ecological process influencing each parameter. Confidence interval is shaded for each model and dashed lines represent *p*-values greater than 0.05 (i.e. the predicted slope was not significantly different from 0).

In contrast, we found a significant increase in krill density (in columns with krill present) in areas of elevated FTLE for the 120 and 240 h integration windows. Krill density was modeled using a log-link function, and our model results are such that the slope describes multiples of change in krill density associated with a one-unit increase in FTLE. For example, the model estimating the geometric mean krill density of cells containing krill in a column at the 120 h integration predicts that an increase of 1 unit of FTLE will increase krill density 2.3 times (slope = 0.822, effect = *e*^0.931^ = 2.275), and for the 240 h integration the increase is 2.6 times (table 2 and figure 3*b*). All three metrics of krill density showed similar increases with increasing integration times (table 2; electronic supplementary material, figure S3).

### (c) Cetacean presence

Blue and humpback whales both showed an increased sighting probability in areas of elevated FTLE, though the temporal scale of the relationship differed between the two species. Blue whales showed a significant positive relationship at all 4 integration windows (table 2 and figure 3*c*), while the relationship for humpback whales was significant at only the 24 and 48 h integration windows (table 2). Sighting probability was modeled using a logit-link function, and our model results are such that the slope describes multiples of change in sighting probability associated with a one-unit increase in FTLE. For example, the model estimating the probability of sighting a blue whale at the 120 h integration predicts that an increase of 1 unit of FTLE will increase sighting probability 3.3 times (slope = 1.2, effect = *e*^1.2^ = 3.32), and for the 240 h integration the increase is 8.3 times (electronic supplementary material, figure S4).

## 4. Discussion

Our findings provide valuable insights into the intricate relationship among oceanographic processes and trophic interactions, including quantifying the role of ocean features on prey distribution and predator occurrence. This study integrated environmental remote-sensing, fisheries acoustics, and visual sightings to describe patterns of predator and prey distribution at scales that are often difficult to measure. The Lagrangian analytical techniques used here may identify transport features that are not apparent using Eulerian analysis methods [75,76] and are particularly useful for studying phenomena such as eddies, fronts and jets, where fluid particles experience complex, time-dependent advection patterns. Our research found that submesoscale processes, which lie between the fine and mesoscales explored in previous studies [17,21,77,78], serve an important aggregative function to the predator-prey scape. These investigations on submesoscale features address a knowledge gap within a continuum of hierarchically nested processes that influence resource distribution, predator–prey interactions, and ultimately the structuring of marine ecosystems.

This study investigated the impact of 4 different integration durations of the FTLE as a proxy for the scale and magnitude of underlying oceanographic processes. By comparing the relationships among multiple interconnected factors and FTLE values obtained for various integration durations, we determined the optimal integration duration that effectively captures trophodynamic features in this region. Our results indicate that the time-scale of processes impacting the near-surface (0–10 m) seawater density, krill density, and cetacean sighting probability is primarily between 2 and 10 days. These results align with the scale of upwelling/relaxation events (1–2 weeks) in the region [21,79,80], as well as the persistence of the majority of krill aggregations (2–10 days) from a regional study in the CCE [32]. Due to the overlap in the calculation of FTLE between the nested integration durations, we may expect some similarity in the resulting relationships, especially among integration durations with higher degrees of overlap (e.g. 24–48, 48–120 and 120–240 h). The patterns we observed however, varied within and between the variables in our analysis, further suggesting that these integration windows are an important tool for teasing apart the scale of processes influencing the ecology of this region. While FTLE is derived from HF Radar data that measures the movements of surface waters to depths of 0.5 to 1.5 m [81,82], our study shows that the depth of influence of aggregative surface current features extends beyond the skin of the ocean to depths of at least 10 m. This finding underscores the utility of Lagrangian metrics in identifying ecological links between surface dynamics and biologically important responses.

Although upwelling was not directly measured in this study, the observed shoaling of isopycnals likely indicates the presence of vertical transport corresponding to recently upwelled waters [65,83]. Previous research on krill predator movements in relation to upwelling dynamics found a similar relationship with seawater density in the presence of an upwelling plume in Monterey Bay [84] and a recent study conducted in the same region found agreement between sea-surface temperature fronts and LCS during summer months [65]. Zooplankton in the CCS has been shown to be distributed on the denser side of fronts [48]. Our results similarly showed that areas of elevated FTLE corresponded to increased seawater density and higher krill density (figure 3 and table 2).

The distribution of prey is driven by a combination of biotic and abiotic factors. While many species of krill are found in the CCS, there are two numerically dominant species, *Euphausia pacifica* and *Thysanoessa spinifera* [29,85]. *E. pacifica* is more abundant particularly offshore (greater than 200 m) but smaller in size than the coastal *T. spinifera* [86]. As a result of the nocturnal period of their diel vertical migration, *E. pacifica* and *T. spinifera* spend roughly 30–50% of their life at or near the surface [87,88] and are subject to horizontal transport driven by surface currents. The distribution of these species, even those found at depth during the day, would thus in part be directly influenced by surface transport pathways (e.g. LCS) that serve to aggregate parcels of water and the organisms within them [89]. Additionally, we would expect krill to be well adapted to take advantage of the aggregative dynamics of a coastal upwelling system to co-locate with their passive, drifting phytoplankton prey [5]. While our study did not distinguish between krill species, the habitat preference, life history and behaviour of each krill species may impact the influence of ocean dynamics and physical forcing, which in turn may influence the scales of patchiness for each species [90]. Further analyses on the species-specific drivers of aggregation may allow a finer-scale understanding of the submesoscale activity of these two important prey species.

At fine scales, krill patches can be homogeneous in terms of size and age-class [91], and adjacent swarms are often made up of cohorts of different—though also homogeneous—size and age-classes [92]. These observations may be reflective of the concept of fluid dynamical niches [93] shown in phytoplankton, in which organismal cohorts remain together over time in parcels of water. These cohorts are formed, for example, by ontogenetic differences in both behaviour and swimming ability, while swarms are believed to be maintained by social interaction [5]. Similar to our findings here, Benoit-Bird *et al.* [21] showed a correlation between increased upwelling activity (i.e. alongshore winds) and increased density within patches of both forage fish and krill, but did not find a relationship to the overall abundance. Additionally, recent work on larval dispersion and aggregative surface current features showed that not all features contained larvae [94]. This aligns with our findings here that not all FTLE features have krill. There is a time lag between primary production following upwelling events and secondary consumer (krill) growth [24], and advective processes lead to krill patches that can be spatially and temporally disassociated from their origin. We did not track the entire trajectory of FTLE features in our study, though future work should investigate the origins of water masses that contain krill or krill prey (e.g. phytoplankton/zooplankton) to determine the conditions that lead to the presence of krill within aggregative features [77,95].

Overall, higher density krill patches represent a more valuable resource for bulk filter-feeding krill predators such as blue and humpback whales. Blue whales have been hypothesized to target the most dense prey patches and most dense regions within a prey patch to optimize their foraging efficiency [33,59]. The link between aggregative surface current features and blue whale feeding performance has been established using a 48 h FTLE integration window [45], which aligns with our results, though we found the strongest relationship at longer integration durations. In contrast, resource partitioning or the ability for humpback whales to prey-switch from krill to fish could help explain the differences observed between the two species in our study. While blue whales are krill obligate foragers, humpback whales are generalists with a mixed diet that depends on relative abundances of fish and krill [96]. Previous research in the region found that blue whales co-located with krill hotspots while the co-occurrence of humpbacks varied [55]. In our study, the relationship between humpback whale sightings and shorter integration windows (24 and 48 h) may correspond to their ability to prey-switch and to target schooling fish, which are a major prey item of humpback whales, with patches more ephemeral than those of krill.

Our data span multiple seasons, years and oceanographic regimes (e.g. Pacific Decadal Oscillation) indicating that FTLE may be a robust metric for identifying dynamic, ephemeral habitat features in the California Current System. The survey effort here occurred between the months of May and September, which coincides with the phenology of krill–predator presence in the region and represents a critical time-period for exploring predator–prey relationships. However, the timing does not fully represent the full range of conditions in the region. For example, the relationships observed here may not hold during strong wind events typically observed during the winter and spring months [30,65,97]. Additionally, while all integration windows aimed to minimize the influence of tides by spanning multiple tidal cycles, the outlet of the San Francisco Bay likely plays a role in the movement of water parcels in our study region. While not investigated here, these effects vary seasonally and are likely to be most pronounced near the bay entrance, and may have a stronger influence on shorter integration durations in our study [98]. Further research in other upwelling regions across a broader range of spatial and temporal scales could help assess whether these results are applicable to other regions of the CCS.

The Central CA region of the CCS that is the focus of this study is an important management area that includes Cordell Bank National Marine Sanctuary (CBNMS) and the Greater Farallones National Marine Sanctuary (GFNMS), as well as a Traffic Separation Scheme (TSS) for shipping activities transiting into and out of San Francisco Bay. Understanding the spatial and temporal distribution of prey species is crucial for implementing effective management strategies for marine predators, such as the establishment of marine protected areas [99,100] or the implementation of vessel traffic controls to reduce whale ship strikes [101]. Furthermore, understanding the relationship between coastal upwelling and krill aggregations enables us to portend the potential impacts of environmental change on these critical ecosystems [102]. Changes in upwelling patterns due to atmospheric shifts could disrupt the availability of forage species (e.g. krill), potentially shifting the distribution of their predators to areas of higher human risk. Anthropogenic impacts on our oceans are increasing [103], with entanglements [104] and ship strikes [55] becoming significant sources of mortality for humpback and blue whales in the California Current. Therefore, uncovering the complex interactions between the physical environment, krill dynamics and whale ecology can aid in their effective management and conservation.

## Data Availability

HF radar data were provided by the U.S. Integrated Ocean Observing System (IOOS) High Frequency Radar Network (HFRNet) and accessed through the Coastal Observing R&D Center (CORDC). Raw *in situ* oceanographic, krill and cetacean data, and raw surface current data have been deposited at Stanford University's digital repository: https://purl.stanford.edu/bk028tz1725. The data and code used in data processing and analyses have been archived at: https://zenodo.org/doi/10.5281/zenodo.10056388 [105]. All processed data and code are also available at the following public GitHub repository: https://github.com/physalus/Krill_and_Lagrangian_Features. Supplementary material is available online [106].
